# Dual pathways from the use of digital technologies to employee creativity: the moderating role of mindfulness

**DOI:** 10.3389/fpsyg.2025.1678376

**Published:** 2025-12-31

**Authors:** Yulong Tu, Shaojie Wang, Lei Lu

**Affiliations:** 1 School of Social and Public Administration, Lingnan Normal University, Zhanjiang, China; 2 School of Business, Hunan University of Humanities, Science and Technology, Loudi, China; 3 School of Psychological and Cognitive Sciences, Beijing Key Laboratory of Behavior and Mental Health, Peking University, Beijing, China

**Keywords:** the use of digital technologies, job flourishing, emotional exhaustion, mindfulness, employee creativity

## Abstract

In the context of increasing digitalization, understanding how the use of digital technologies affects employee creativity is essential for promoting enterprise innovation. However, existing research on the impact of the use of digital technologies on employee creativity is inconsistent. Even when scholars acknowledge these conflicting findings, few have investigated the underlying mechanisms behind these differences. To fill this gap, this study proposes a dual-pathway model, considering job flourishing and emotional exhaustion as mediators and mindfulness as a moderator, based on the Conservation of Resources Theory. Analyzing 757 valid samples in China using multiple linear regression and Bootstrap tests, the results indicate that the use of digital technologies can enhance job flourishing and boost employee creativity, but it can also lead to emotional exhaustion, which in turn reduces employee creativity. Mindfulness is found to strengthen the link between the use of digital technologies and job flourishing, while weakening the connection between the use of digital technologies and emotional exhaustion. Additionally, mindfulness can amplify the indirect effect of the use of digital technologies on employee creativity through job flourishing and diminish its indirect effect via emotional exhaustion. These findings help enterprises maximize benefits and mitigate the potential negative effects of using digital technologies to support sustainable innovation.

## Introduction

Employee creativity involves generating novel and useful ideas by individuals within an organization. It is essential for promoting enterprise development, as it significantly impacts an organization’s competitiveness and capacity for innovation (Gong et al., 2009). Digital technologies encompass a range of potent, accessible, and potentially transformative innovations, including social media, mobile apps, cloud computing, analytics, the Internet of Things, cognitive computing, and biometrics (Vermesan and Friess, 2022). The use of digital technologies influences how employees work (Fleischer and Wanckel, 2024; Siyal et al., 2023) and shapes the work environment (Cahen and Borini, 2020; Kaltenegger et al., 2020; Tekic and Koroteev, 2019), which is related to employee creativity (Cetindamar-Kozanoglu and Abedin, 2021; Korzynski et al., 2019).

However, the existing literature also reveals inconsistent effects of using digital technologies (Marsh et al., 2022). Some scholars have shown that the use of digital technologies can improve the quick transfer (Chatterjee et al., 2023; Huu, 2023) and sharing of information, allowing employees to access and combine knowledge resources more rapidly (Creswell, 2017; Nikou et al., 2022; Wang et al., 2022). Employees can quickly analyze and extract insights from large amounts of data through digital technologies to identify potential problems and opportunities (Vodă et al., 2022; Yang et al., 2017). Using digital technologies like AI (Ameen et al., 2022) and digital platforms (Cassetta et al., 2020; Wang et al., 2020) offers employees more convenient (Berente et al., 2021; Oldham and Da Silva, 2015) and efficient communication methods (Attaran et al., 2019; Deng et al., 2023; Janse van Rensburg et al., 2022) and collaboration platforms (Pink et al., 2017), giving employees greater flexibility and helping them better manage their tasks (Bondanini et al., 2020; Bousinakis and Halkos, 2021; Tortorella et al., 2021).

While some scholars argue that the use of digital technologies causes employees to feel anxious about dedicating more attention to their professional development (Fiedler et al., 2021; Pfaffinger et al., 2020), a lower-skilled workforce may increasingly face displacement by digital technologies and concerns about unemployment. This, in turn, can reduce their motivation and effort at work (Orlandi et al., 2024). Instant messaging provides access to work information at any time, but can hinder efficiency, causing fatigue and reduced productivity (Mulki et al., 2006). Remote workers often work longer hours, which can boost productivity but also lead to feelings of loneliness and exhaustion (Chesley, 2014; Polly et al., 2021; Sonnentag et al., 2010). Overuse of digital technologies can cause cognitive overload (Bunjak et al., 2021), leading to burnout (Montreuil et al., 2022; Stich et al., 2015), cyberloafing (Aciksoz, 2024; Henle and Blanchard, 2008; Lim, 2002; Puranik et al., 2019), and reduced creativity among workers (Bunjak et al., 2021). Although some scholars have noted that the use of digital technologies has a double-edged effect on employee creativity, there is a lack of exploration into the internal mechanisms underlying this double-edged effect. Therefore, we believe that the impact of using digital technologies on employee creativity is complex. Although some scholars have noted this, there has been little exploration of the internal mechanisms underlying this dual effect.

The research gap is important because digital transformation is crucial for an enterprise’s competitiveness, but it involves changes related to employees. If employees view digital technologies only as sources of technical pressure and burnout, the likelihood of successful digital transformation becomes uncertain (Ertiö et al., 2024). Therefore, digital transformation is not just about investing in digital technologies (Jan et al., 2022); understanding how the use of digital technologies impacts employees is even more critical. This understanding enables enterprises to intervene early and support successful digital transformation.

So, to fill this gap, we aim to develop a dual-pathway model that explains how the use of digital technologies differently affects employee creativity. By combining job flourishing and emotional exhaustion, we introduce a resource gain spiral and loss spiral perspective based on the Conservation of Resources (COR) theory (Hobfoll et al., 2003) to reconcile previous contradictory findings. According to COR theory, a gain spiral can lead to an increase in resources, promote job flourishing, and stimulate creativity. Conversely, a loss spiral can evoke negative emotions, lead to emotional exhaustion, and diminish creativity. We believe that whether the use of technology has a positive impact on employee creativity depends on whether digital technology can be effectively leveraged as a resource in the workplace.

Meanwhile, our focus is directed towards employee mindfulness to comprehend how individual differences in the use of digital technologies influence employee creativity. Mindfulness, defined as conscious attention (Gajda and Zbierowski, 2023; Loucks et al., 2022), affects attitudes and behaviors, including well-being, performance, workplace aggression, anti-consumption, and prosocial actions (Lyddy et al., 2021; Zheng et al., 2023; Liang et al., 2018; Daniel et al., 2024; Kil et al., 2021; De Vibe et al., 2013). We propose that mindfulness may serve as a moderating factor in employees’ psychological states, thereby impacting their creativity, particularly through mechanisms such as job flourishing and emotional exhaustion.

## The theoretical basis and development of hypotheses

### The mediating role of job flourishing and emotional exhaustion

COR theory suggests that individuals use available resources to achieve work goals and strive to maintain valuable assets (Hobfoll, 2001; Hobfoll and Wells, 1998). Differences in resource allocation can lead to two distinct paths for employees: a gain spiral and a loss spiral (Saniuk et al., 2023). The gain spiral occurs when the use of digital technologies enables employees to access extensive business data, allowing them to acquire essential knowledge, skills, and information for their work (Hakanen et al., 2008; Hobfoll, 2002). Employees with more work resources feel more energetic and engaged in their tasks. This engagement encourages them to pursue new knowledge and skills, leading to job flourishing. Job flourishing typically refers to situations where employees are given opportunities for growth, learning, and development within their roles. These opportunities allow them to acquire valuable knowledge and skills (Halbesleben et al., 2014). As employees accumulate these resources, they not only enhance their professional competencies but also elevate their self-efficacy or confidence in their capacity to accomplish tasks and resolve issues (Erum et al., 2020; Keyes, 2020). This increase in confidence fosters greater openness and adaptability when confronting novel challenges (Hatch and Dyer, 2004), thereby stimulating their intrinsic motivation and curiosity, which makes them more inclined to explore and pursue new opportunities (Hendrawan et al., 2024), ultimately leading to an enhancement of their creativity.

Conversely, organizations undergoing digital transformation frequently face resource limitations, including a lack of adequate personnel, funding, outdated technological systems, and insufficient infrastructure, as documented by Carnevale and Hatak (2020). These shortages often necessitate that existing employees assume additional responsibilities, requiring them to fulfill their original duties while managing projects related to digital technologies, as noted by Marion and Fixson (2021). Consequently, employees often oscillate between various tasks (MacDonald, 2003), which elevates their workload and stress levels. During the digital transformation process, employees must dedicate considerable time and effort to acquiring new skills, which can induce anxiety regarding their competence (Cetindamar-Kozanoglu and Abedin, 2021). If these issues are not addressed over time, employees may experience emotional exhaustion, characterized by feelings of depletion, fatigue, helplessness, anxiety, and diminished self-esteem, thereby reducing their psychological resources (Welp et al., 2015). Such emotional exhaustion often results in individuals’ attention being primarily consumed by anxiety itself, impairing their capacity to allocate adequate cognitive resources to creative thinking (Mulki et al., 2006). Employees experiencing chronic anxiety may develop rigid cognitive patterns that overly emphasize potential risks and threats while neglecting opportunities and possibilities. This rigidity limits cognitive flexibility, hindering the ability to move beyond traditional frameworks and develop innovative solutions and ideas. Additionally, emotional anxiety can obstruct employees’ social interactions, making it difficult to establish effective communication and collaborative relationships with others (Mulki et al., 2006). This restriction reduces opportunities for sharing creative ideas and consequently lowers overall employee creativity. Therefore, the study argues that:

*H1:* Job flourishing mediates the relationship between the use of digital technologies and employee creativity.

*H2:* Emotional exhaustion mediates the relationship between the use of digital technologies and employee creativity.

### The moderation role of mindfulness

Mindfulness is a practice involving intentional and nonjudgmental attention (Brown and Ryan, 2003), which encompasses self-regulation of focus on immediate experiences and cultivating an open, accepting attitude towards such experiences (Creswell, 2017). It is regarded as a cognitive resource that facilitates employees’ ability to concentrate on their work in the present moment (Glomb et al., 2011; Gunasekara and Zheng, 2019), thereby contributing to increased work productivity (Henriksen et al., 2020). According to COR theory, individuals possessing abundant resources are more inclined to self-expand and experience heightened motivation. Zivnuska et al. (2016) demonstrate how mindfulness within the workplace can aid employees in developing valuable resources, which in turn can enhance well-being by reducing psychological distress and augmenting job satisfaction. Ngo et al. (2020) indicate that mindfulness at work can enhance work performance. Goyal and Sharma (2024) suggest that mindfulness enhances employees’ work engagement both directly and indirectly. Consequently, mindfulness can help employees respond more effectively to job challenges. Research by Yan et al. (2024) revealed that mindfulness at work may alleviate workplace loneliness among IT professionals. Overall, mindfulness constitutes a personal resource that intentionally focuses on the present moment, undistracted by external disturbances. We posit that it fosters the positive effects of the use of digital technologies on job flourishing.

However, the use of digital technologies can require significant time and effort from employees (Kim and Beehr, 2020). Research indicates that digital technology use can become problematic for some employees, leading to excessive time spent online. This dependence on digital technology may disrupt their work-life balance (Dén-Nagy, 2014), increase burnout due to the constant influx of information (Day et al., 2010), and raise concerns about job security (Ferguson et al., 2016), ultimately leading to emotional exhaustion (Becker et al., 2019). Mindfulness enhances employees’ ability to detach themselves, freeing them from interference and reducing negative emotions (Walsh et al., 2022). Studies show that mindfulness-based interventions can alleviate symptoms of various mental health issues, like depression, anxiety, and post-traumatic stress disorder (Hofmann et al., 2010). Such interventions can also improve attentional control (Verhaeghen, 2021). Therefore, mindfulness can help regulate emotional and mental health, improve focus, and lessen anxiety that affects sleep (Cao et al., 2022), thereby reducing emotional exhaustion, enhancing social skills and adaptability at work, alleviating resource shortages, and shaping job roles (Hur et al., 2024). Tang et al. (2019) argues that individuals with high mindfulness levels perceive reality through observation, manage their control, and boost their self-confidence and self-evaluation. Kim et al.'s (2024) research found that the group with problematic smartphone use exhibited lower mindfulness than the group with normal use. In this context, mindfulness enables employees to become more resilient in the face of challenges. Therefore, this study suggests that:

*H3:* Mindfulness positively moderates the positive relationship between the use of digital technologies and job flourishing. The effect is stronger when mindfulness is high.

*H4:* Mindfulness buffers the positive relationship between the use of digital technologies and emotional exhaustion. The effect is weaker when mindfulness is high.

### An integrated model

Hypothesis 1 suggests that job flourishing mediates the relationship between the use of digital technologies and employee creativity. Additionally, Hypothesis 3 proposes that mindfulness moderates the impact of the use of digital technologies on job flourishing. Based on these assumptions, we propose that employees’ mindfulness moderates the mediating effect of the use of digital technologies on employee creativity through job flourishing. Similarly, we believe that employees’ mindfulness also moderates the mediating effect of the use of digital technologies on employee creativity via emotional exhaustion. Therefore, considering both the mediation and moderation hypotheses, the study argues that:

*H5:* Mindfulness moderates the mediated relationship between the use of digital technologies and employee creativity via job flourishing.

*H6:* Mindfulness moderates the mediated relationship between the use of digital technologies and employee creativity via emotional exhaustion.

**Figure 1:**
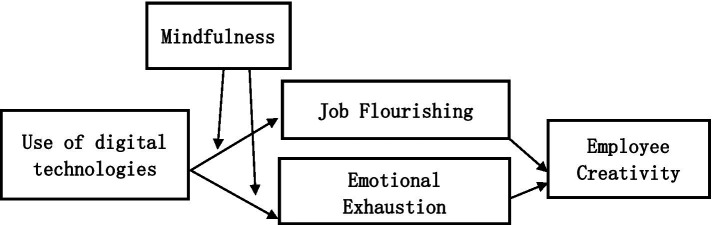
The conceptual research model.

The proposed conceptual research model is as follows (Figure 1):

## Methodology

### Sampling and data collection

The recruitment of questionnaire respondents was posted in a community discussion on the local Talent Network. This was done because the community platform of the talent market attracts many employees, HR managers, and job seekers, especially those interested in developing and using digital technologies. The announcement clearly stated that the questionnaire would be used solely for scientific research and not for commercial purposes. All participants would remain anonymous and keep their personal information confidential. This research was approved by the Ethical Committee of Lingnan Normal University (approval number: S20250601). The questionnaire was designed to be completed in two rounds, with a two-week gap between each. Participants who finished the survey could receive 5 RMB as compensation. Those interested in participating can join the QQ group (a popular social media platform in China) for more details and to check the deadline.

Between February 2025 and March 2025, links to the questionnaire were shared in the QQ group. Participants were required to include their phone numbers on each questionnaire to facilitate matching responses from both rounds and to ensure the timely distribution of rewards. A total of 1,200 people joined the group. We utilized a two-wave online questionnaire approach to avoid common-method bias (Podsakoff et al., 2003). The first questionnaire included control, independent, mediating, and moderating variables. At Time 1, we collected 955 responses. After four weeks, only the dependent variable was retained, pairing the Time 2 responses with those from Time 1 to form a complete dataset. After excluding invalid questionnaires that were either completed carelessly or possibly duplicated, we obtained 757 valid responses, resulting in a recovery rate of 63.08%. As shown in Table 1, 46.9% of the respondents were female, and 71.6% of the employees fell within the age range of 20 to 40. Most respondents (66.4%) held a bachelor’s degree or higher. The distribution of respondents by industry was as follows: manufacturing (35.7%), information and communication (32.6%), education (20.6%), and other sectors (11.1%).

**Table 1:** Demographic description of respondents.

Demographics	No. of respondent	Percent (%)
Gender		
Male	402	53.1
Female	355	46.9
Age group		
20–30	273	36.1
31–40	269	35.5
over 40 years	215	28.4
Education		
Specialist or under 254		33.6
Bachelor	341	45.0
Master	153	20.2
Doctor	9	1.2
Tenure in current organization		
Less than 1 year	91	12.0
1–3 years	245	32.4
4–5 years	195	25.8
6–10 years	132	17.4
Over 10 years	94	12.4
Industry		
Manufacturing	270	35.7
Information and communication	247	32.6
Education	156	20.6
Others	84	11.1

### Measure

All key variables were rated using a five-point Likert scale (1 = strongly disagree to 5 = strongly agree).

#### The use of digital technologies

Existing scales mostly target single digital tools and do not cover the multidimensional use of technologies such as IoT, big data, and cloud computing, which are central to this study, thereby failing to fully reflect employees’ technological scenarios during digital transformation. According to affordance theory (Hutchby, 2001), the use of digital technologies at the enterprise level creates opportunities and constraints for individuals. Hence, enterprise-level use serves as a proxy for individual use, measured by an 8-item scale from Zahoor et al. (2024). The respondents were asked to evaluate the extent to which their enterprises adopted various technologies, including the Internet of Things, social media, mobile computing, electronic commerce, digital platforms, business intelligence, big data analytics, and cloud computing. The Cronbach’s alpha coefficient for this survey was 0.886.

#### Job flourishing

Job flourishing was measured using a 10-item scale by Porath et al. (2012). An example item was “I often study.” Cronbach’s alpha coefficient was 0.901.

#### Emotional exhaustion

Emotional exhaustion was assessed using a 5-item scale, as employed by Li and Shi (2023). A sample was “Working all day is stressful for me.” Cronbach’s alpha coefficient was 0.882.

#### Mindfulness

The measurement of mindfulness in this study focuses on “mindfulness in the workplace.” Its goal is to study how employees’ mindfulness moderates the impact of the use of digital technologies on their creativity in the workplace. The scale selection is based on the works of Zheng et al. (2023), comprising a six-item scale. EFA was conducted via principal axis factoring and orthogonal varimax rotation; the KMO value was 0.83, and Bartlett’s test of sphericity was significant. All item factor loadings exceeded 0.7, with a cumulative variance explained of 63.24%, indicating that the scale is suitable for this study. One example is “I focused my attention on the present task.” The Cronbach’s alpha coefficient for this measure was 0.899.

#### Employee creativity

Employee creativity was assessed using a 7-item scale developed by Gong et al. (2009). A sample was “I often develop creative custom-made product/service packages for clients.” Cronbach’s alpha coefficient was 0.882.

#### Control variables

Prior studies have indicated that gender, age, education, tenure in the current organization, and industry are directly linked to employee creativity (Oldham and Cummings, 1996). Therefore, these variables serve as control variables in this study.

## Results

### Common method variance

The questionnaires were collected in two waves to address common method variance, as recommended by Podsakoff et al. (2003). Additionally, the Harman single-factor test was used to test the data, which revealed five common factors with eigenvalues greater than one. The results indicated a variation of 21.45% against a single excluded feature, which is significantly lower than the 50% limit (Hair et al., 1998). We also carried out a latent method factor analysis, following the procedure outlined by Podsakoff et al. (2003). We loaded the method factor on all indices. Analytical results revealed that the six-factor measurement model, which included the common method factor and critical variables (Chi2/df = 1.554, RMSEA = 0.027, CFI = 0.979, GFI = 0.978), did not exhibit a better fit to the data than the five-factor measurement model. Therefore, the CMV of this study presents a manageable risk to the validity of our results.

### Confirmatory factor analyses

We evaluated our measurement model using confirmatory factor analysis. The model proposed in our study included five variables: the use of digital technologies, mindfulness, job flourishing, emotional exhaustion, and employee creativity, which are grouped under Model 1. We compared a hypothesized 4-factor model against nested 3-factor, 2-factor, and 1-factor models. The fit indices indicated that our hypothesized model provided the best fit (χ^2^ (454) = 705.981, CFI = 0.979, TLI = 0.977, RMSEA = 0.027), as shown in Table 2. Our analysis verified that the proposed measure adequately tested the hypothesized relationship. We performed tests to assess convergent validities (CR and AVE) and discriminant validities (MSV and ASV). The results in Table 3 indicate that the AVE value for all constructs exceeded the threshold of 0.50, and the CR value was above 0.70. Additionally, all constructs’ MSV and ASV values were lower than their respective AVE values. Based on these findings, we can conclude that the study exhibits good convergent and discriminant validity.

**Table 2:** Results of CFAs: comparison of measurement models.

Model	χ^2^	df	χ2/df	RMSEA	CFI	TLI
Baseline model	705.981	454	1.555	0.027	0.979	0.977
Four-factor model: UDT+MIN, JF, EE, EC	3171.695	458	6.925	0.089	0.778	0.760
Four-factor model: UDT+ JF, MIN, EE, EC	2804.446	458	6.123	0.082	0.808	0.792
Four-factor model: UDT+ EE, MIN, JF, EC	2290.385	458	5.001	0.073	0.850	0.838
Four-factor model: UDT, MIN, EE+JF, EC	2760.379	458	6.027	0.082	0.812	0.796
Three-factor model: UDT, MIN+JF+EE, EC	5179.341	461	11.275	0.116	0.614	0.585
Two-factor model: UDT+MIN+JF+EE, EC	7130.318	463	15.400	0.138	0.455	0.416
One-factor model: UDT+MIN+JF+EE+EC	8801.273	464	18.968	0.154	0.319	0.272

UDT = Use of digital technologies; MIN = Mindfulness; JF = Job flourishing; EE = Emotional emotion; EC = Employee creativity.

**Table 3:** Factor loading estimates.

Constructs	Item	Loading	CR	AVE	MSV	ASV
Use of digital technologies	8	0.736 ~ 0.765	0.886	0.565	0.127	0.09
Mindfulness	6	0.713 ~ 0.832	0.900	0.600	0.012	0.012
Job flourishing	10	0.685 ~ 0.738	0.901	0.504	0.118	0.063
Emotional exhaustion	5	0.740 ~ 0.734	0.883	0.601	0.048	0.062
Employee creativity	7	0.690 ~ 0.822	0.886	0.565	0.118	0.083

CR = Composite Reliability, AVE = Average Variance Extracted, MSV = Maximum Shared Variance, ASV = Average Shared Variance.

### Descriptive statistics

The study’s descriptive and correlation analyses are presented in Table 4. The use of digital technologies was significantly and positively associated with job flourishing (*r* = 0.244, *p* < 0.01). The use of digital technologies was also significantly and positively associated with emotional exhaustion (*r* = 0.357, *p* < 0.01). Job flourishing significantly and positively correlated with employee creativity (*r* = 0343, *p* < 0.01). Emotional exhaustion was significantly and negatively correlated with employee creativity (*r* = −0.218, *p* < 0.01). Similarly, the use of digital technologies was significantly and positively correlated with employee creativity (*r* = 0.287, *p* < 0.01). These preliminary results align with our hypotheses.

**Table 4:** Means, standard deviations, and correlations (*N* = 757).

Variable	M	SD	1	2	3	4	5	6	7	8	9
1. Age	1.557	0.497									
2. Gender	2.923	0.800	−0.002								
3. Education	2.350	0.998	0.057	−0.079^*^							
4. Industry	1.793	0.673	−0.022	0.010	−0.112^**^						
5. Organizational tenure	2.859	1.207	0.010	−0.036	−0.101^**^	0.091^*^					
6. UDT	3.207	1.146	0.062	0.042	−0.017	0.073^*^	−0.044				
7. JF	3.382	0.973	0.052	0.019	−0.016	0.008	−0.017	0.244^**^			
8. EE	3.122	1.157	−0.009	−0.007	−0.013	0.073^*^	−0.006	0.357^**^	−0.036		
9. Mindfulness	3.772	1.018	−0.037	0.025	−0.023	−0.009	0.004	−0.034	0.110^**^	0.000	
10. EC	3.436	1.065	0.056	0.016	0.019	−0.005	−0.022	0.287^**^	0.343^**^	−0.218^**^	0.023

* *p* < 0.05. ** *p* < 0.01. UDT = Use of digital technologies; MIN = Mindfulness; JF = Job flourishing; EE = Emotional emotion; EC = Employee creativity.

### Hypotheses testing

A hierarchical regression analysis was conducted to test hypotheses H1 to H4. This approach helps understand how different factors contribute to the outcome variable while controlling for other variables. The results of the hierarchical regression model are presented in Table 5, along with standardized beta values. As reported in Model 1, after controlling for all variables in Step 1, we regressed the mediation variable (job flourishing) on the independent variable (the use of digital technologies) in Model 8. The results showed that the use of digital technologies was positively related to job flourishing (𝛽 = 0.242, *p* < 0.001), thus meeting the first requirement for mediation. We found that after controlling for the effects of gender, age, education, tenure, and industry in Model 1, the effect of job flourishing on employee creativity was significant (𝛽= 0.341, *p* < 0.001) in Model 2. These results met the second requirement for mediation. We then entered job flourishing into Model 5 to test its possible mediating effect on the relationship between the use of digital technologies and employee creativity. We found job flourishing to be significantly related to employee creativity (𝛽= 0.289, *p* < 0.001), whereas the coefficient of the use of digital technologies was reduced in size but remained significant (𝛽= 0.217, *p* < 0.001). Thus, Hypothesis 1 was verified. Similarly, the use of digital technologies was significantly related to emotional exhaustion (𝛽 = 0.357, *p* < 0.001) in Model 10. Emotional exhaustion was significantly related to employee creativity (𝛽 = −0.219, *p* < 0.001) in Model 4. Then, we entered emotional exhaustion into Model 6 to test its possible mediating effect on the relationship between the use of digital technologies and employee creativity. We found emotional exhaustion to be significantly related to employee creativity (𝛽 = −0.367, *p* < 0.001), which suggested the use of digital technologies could affect emotional exhaustion and then negatively affect employee creativity. Hypothesis 2 was thus supported.

**Table 5:** Hierarchical regression analysis results of emotional exhaustion.

Dependent variable	EC				JF					EE	
Model	M1	M2	M3	M4	M5	M6	M7	M8	M9	M10	M11
Control variables											
Gender	0.055	0.037	0.036	0.054	0.026	0.026	0.053	0.038	0.043	−0.030	−0.032
Age	0.017	0.011	0.006	0.015	0.004	−0.002	0.017	0.008	0.006	−0.022	−0.024
Education	0.015	0.021	0.019	0.014	0.023	0.019	−0.02	−0.015	−0.017	−0.001	0.005
Industry	−0.021	−0.014	−0.006	−0.024	−0.004	−0.004	−0.02	−0.007	−0.009	0.005	0.008
Tenure	−0.001	−0.004	−0.023	0.015	−0.020	−0.006	0.008	−0.010	−0.003	0.045	0.038
Independent variable											
UDT			0.287***		0.217***	0.417***		0.242***	0.247***	0.357***	0.356***
Mediating variable											
JF		0.341***			0.289**						
EE				−0.219***		−0.367***					
Moderation variable											
Mindfulness									0.119***		0.013
Interactive effect											
UDT*Mindfulness									0.110**		−0.140***
R^2^	0.004	0.346	0.085	0.052	0.163	0.202	0.004	0.061	0.088	0.131	0.151
ΔR^2^	0.004	0.116	0.081	0.048	0.078	0.117	0.004	0.058	0.084	0.126	0.145

*N* = 757, *p < 0.05, **p < 0.01, ***p < 0.001 (two-tailed tests). UDT = Use of digital technologies; MIN = Mindfulness; JF = Job flourishing; EE = Emotional emotion; EC = Employee creativity.

**Figure 2:**
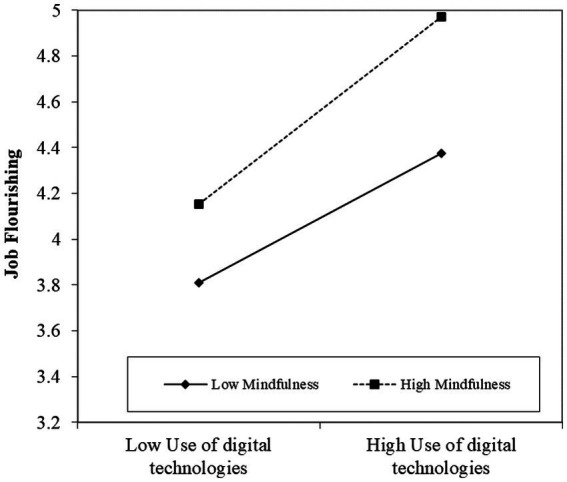
Interaction of use of digital technologies and mindfulness on job flourishing.

**Figure 3:**
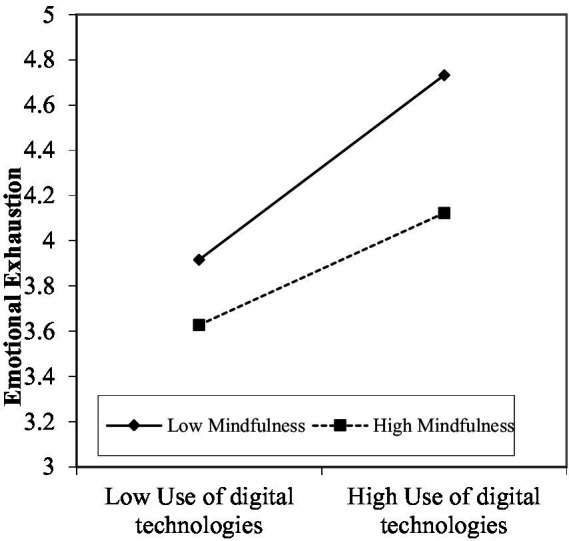
Interaction of use of digital technologies and mindfulness on emotional exhaustion.

To test the moderation model of H3 and H4. Job flourishing was first set as the dependent variable, and the control variables, the use of digital technologies, and the moderator variable (mindfulness) were introduced. Finally, a centralized interaction term for the use of digital technologies and mindfulness was added in Model 9. We found that the interactive effect of the use of digital technologies and mindfulness on job flourishing (𝛽 =0.110, *p* < 0.001) was significant. We plotted the interactive effect on job flourishing in Figure 2. Supporting Hypothesis 3, simple slope analyses revealed that the use of digital technologies was strongly related to job flourishing among employees who exhibited higher levels of mindfulness compared to those with lower levels. The effect of using digital technologies on job flourishing was more strongly related when employees’ mindfulness was higher than when it was lower. Moreover, we also found that the interactive effect of the use of digital technologies and mindfulness on emotional exhaustion (𝛽 = −0.140, *p* < 0.001) was significant in Model 11. We plotted the interactive effect on emotional exhaustion in Figure 3. Simple slope analyses revealed that the use of digital technologies was less strongly related to emotional exhaustion among employees who exhibited higher levels of mindfulness compared to those with lower levels. Thus, H4 was supported.

We used PROCESS on 5,000 Bootstrap samples to examine the moderated mediation of H5 and H6, according to the recommendations of Preacher and Hayes (2008). The investigation was performed at a low and high degree of mindfulness. Table 6’s results indicated that the indirect effects of the use of digital technologies on employee creativity through job flourishing were significant at a high level of employees’ mindfulness (0.087, Boot 95% CI [0.058, 0.122], excluding zero) and a low level of employees’ mindfulness (0.046, Boot 95% CI [0.017, 0.080], excluding zero). The indirect effects of the use of digital technologies on employee creativity via job flourishing were stronger for employees with high-level mindfulness than those with low mindfulness. In addition, the difference between the two groups was significant (0.041, Boot *SE* = 0.187, and Boot 95% CI [0.005, 0.078]). Hence, hypothesis 5 was supported. Similarly, the indirect effects of the use of digital technologies on employee creativity via emotional exhaustion were weaker for employees with high-level mindfulness (−0.085, Boot 95% CI [−0.113, −0.049]) than for those with low mindfulness (−0.158, Boot *SE* = 0.019, Boot 95% CI [−0.196, −0.121]). The difference between the two groups was significant (0.077, Boot *SE* = 0.021, and Boot 95% CI [0.038, 0.121]). Thus, hypothesis 6 was supported.

**Table 6:** Bootstrap test of moderation mediation effects.

Indirect effect	Effect	BootSE	Boot 95% LLCI	Boot 95% ULCI
Path	Use of digital technologies → Job Flourishing → Employee Creativity			
Low	0.046	0.016	0.017	0.080
High	0.087	0.016	0.058	0.122
Difference	0.041	0.187	0.005	0.078
path	Use of digital technologies → Emotional Exhaustion → Employee Creativity			
Low	−0.158	0.019	−0.196	−0.121
High	−0.085	0.016	−0.113	−0.049
Difference	0.077	0.021	0.038	0.121

*N* = 757, BootSE = Bootstrap Standard Error, Boot 95%CI = Bootstrap 95% Confidence Interval, Bootstrap samples = 5000.

## Discussion

This paper develops a dual pathway model based on COR, showing that the use of digital technologies influences employee creativity through job flourishing and emotional exhaustion. The use of digital technologies enhances communication and resources, boosting creativity, but also causes stress from techno-uncertainty and upgrades, leading to emotional exhaustion and a decline in creativity. It highlights mindfulness as a moderator, which can enhance the gain path and buffer the loss path, thereby optimizing the overall effect of the use of digital technologies. The study explains this complex, paradoxical relationship and deepens understanding of the use of digital technologies in the workplace.

### Theoretical contributions

The current research offers vital theoretical contributions to the existing literature by exploring the relationship between the use of digital technologies and employee creativity.

First, this study breaks through the theoretical framework of a single effect, providing a theoretical basis for understanding the “double-edged sword” effect of the use of digital technologies in the workplace. It explicitly proposes that the use of digital technologies has a dual impact on employee creativity through either job flourishing or emotional exhaustion, revealing that the influence of the use of digital technologies on employee creativity is not simply promoting or inhibiting, but rather the result of a balance of two forces.

Secondly, the study introduces “job flourishing” and “emotional exhaustion” as mediators, expanding the understanding of the mechanism by which the use of digital technologies affects employees’ creativity through the gain spiral and the loss spiral based on COR theory. “Job flourishing” connects “technology empowerment-resource accumulation” to “employee creativity through the gain spiral. “Emotional exhaustion” links “technology pressure-resource loss” to the obstruction of employee creativity through a loss spiral. Verifying this mechanism clarifies the “psychological black box” through which the use of digital technologies impacts employee creativity.

Thirdly, the study shows that mindfulness plays a dual regulatory role in how the use of digital technologies affects employee creativity, expanding research on individual differences. Mindfulness boosts employees’ sense of empowerment from digital technology, strengthening the positive link between job flourishing and creativity. It also reduces overreactions to digital stress, weakening the negative impact of emotional exhaustion on creativity. This challenges the view that regulatory variables have only a single impact, illustrating that individual traits can act as both buffers and amplifiers in the relationship between the use of digital technologies and employee creativity.

### Managerial implications

This study has several managerial implications. Firstly, when implementing digital technology, enterprises should focus on strengthening the development of psychological resources that support their efforts. Since mindfulness can effectively manage the effects of digital technology use, enterprises should incorporate mindfulness training into their employee development programs. Through regular workshops and training sessions, employees can enhance their concentration and emotional regulation skills, enabling them to maintain a steady mindset and efficiently manage multiple tasks, thereby establishing a psychological foundation for creativity.

Secondly, work design must consider the limitations of the use of digital technology. Since the use of digital technologies can pose risks such as emotional exhaustion, enterprises should establish usage guidelines to prevent employees from multitasking and overexertion. For example, a “feedback platform for the use of digital technologies” allows employees to report unreasonable rules or excessive interference. Dedicated personnel regularly review feedback and adjust usage norms accordingly, making work design more aligned with employees’ needs and encouraging cooperation in the use of digital technologies.

Finally, leadership development should keep pace with current trends and incorporate digital literacy as a key component. Managers need the ability to recognize signs of technology overload and develop management skills to strike a balance between efficiency and creativity. Enterprises should make “digital leadership” a central part of management training, helping managers drive digital transformation while safeguarding the team’s psychological well-being and protecting employees’ creativity from the negative effects of the use of digital technologies.

## Limitations and future research

While this study has achieved its specific objectives, some limitations must be acknowledged. First, this study includes educational level as a control variable, which correlates with digital technology literacy. Higher education often indicates stronger skills and greater acceptance of digital technology. However, digital literacy pertains to mastery of digital tools. Controlling for education reduces confounding, but some employees may still differ in their digital literacy, which can affect psychological responses and outcomes. Future research could consider digital literacy as a moderating variable.

Second, the sample size is large, but all participants are based in China; this geographic limitation may limit the findings’ generalizability. Future research should adopt a cross-national, multi-regional sampling design, selecting enterprise samples along cultural dimensions based on Hofstede’s theory to explore how cultural differences influence the impact of digital technology on employee creativity. Meanwhile, the current sample, limited to online interviews, may introduce selection bias, favoring more digitally adept employees or those with specific levels of job satisfaction. Future studies should use a “multi-channel” approach that combines online and offline methods to obtain a more representative sample. Additionally, including work satisfaction as a control or moderator could reveal its interaction with digital tech use, mindfulness, and other factors affecting employee creativity.

Third, the sample includes manufacturing (35.7%), information and communication (32.6%), education (20.6%), and other fields, but lacks a detailed analysis of industry characteristics like technology, labor, or knowledge intensity as moderators. For example, employees in information may have higher digital proficiency than those in manufacturing, and emotional exhaustion risks from the use of digital technologies in education may differ from those in manufacturing. Future research should consider “industry type” as a moderator to examine how the use of digital technologies affects their creativity across industries, thereby improving industry applicability.

Fourth, this study assesses the use of digital technologies at the enterprise level, guided by the affordable theory. Since enterprises engage in activities that showcase digital deployment and resource allocation, this offers a straightforward variable for the research framework. However, this method primarily captures organizational decisions and resource allocation, rather than individual usage motivations or impacts. Future research should develop tools to measure the use of digital technologies at the individual level to yield more accurate insights.

Finally, this study focuses solely on the moderating effect of employee mindfulness on the relationship between the use of digital technologies and employee creativity, potentially neglecting the impact of individual differences on this relationship, such as the Big Five personalities having different levels of digital technology (Diller et al., 2020), which is likely to be different in the process of the use of digital technologies on employee creativity. Thus, future researchers have numerous opportunities to contribute to this field and enhance our understanding of the use of digital technologies and their influence on employee creativity in the workplace.

## Data Availability

The original contributions presented in the study are included in the article/supplementary material, further inquiries can be directed to the corresponding author.

## References

[ref1] Aciksoz S. (2024). A meta-analytic investigation of cyberloafing. Career Dev. Int. 22, 546–564. doi: 10.1108/CDI-08-2017-0142

[ref2] Ameen N. Sharma G. D. Tarba S. Rao A. Chopra R. (2022). Toward advancing theory on creativity in marketing and artificial intelligence. Psychol. Mark. 39, 1802–1825. doi: 10.1002/mar.21699

[ref3] Attaran M. Attaran S. Kirkland D. (2019). The need for a digital workplace: increasing workforce productivity in the information age. Int. J. Enterp. Inf. Syst. 15, 1–23. doi: 10.4018/IJEIS.2019010101

[ref4] Becker W. J. Belkin L. Y. Conroy S. A. Tuskey S. (2019). Killing me softly: organizational e-mail monitoring expectations’ impact on employee and significant other well-being. J. Manage. 47, 1024–1052. doi: 10.1177/0149206319890655

[ref5] Berente N. Gu B. Recker J. Santhanam R. (2021). Managing artificial intelligence. MIS Q. 45, 1433–1450. doi: 10.25300/MISQ/2021/16274

[ref6] Bondanini G. Giorgi G. Ariza-Montes A. Vega-MuÃOZ A. Andreucci-Annunziata P. (2020). Technostress: the dark side of technology in the workplace. A scientometric analysis. Int. J. Environ. Res. Public Health 17:8013. doi: 10.3390/ijerph17218013 33143270 PMC7662498

[ref7] Bousinakis D. Halkos G. (2021). Creativity as the hidden development factor for organizations and employees. Econ. Anal. Policy 71, 645–659. doi: 10.1016/j.eap.2021.07.003

[ref8] Brown K. W. Ryan R. M. (2003). The benefits of being present: mindfulness and its role in psychological well-being. J. Pers. Soc. Psychol. 84, 822–848. doi: 10.1037/0022-3514.84.4.822, 12703651

[ref9] Bunjak A. Černe M. Popovič A. (2021). Absorbed in technology but digitally overloaded: interplay effects on gig workers’ burnout and creativity. Inf. Manag. 58:103533. doi: 10.1016/j.im.2021.103533

[ref10] Cahen F. Borini F. M. (2020). International digital competence. J. Int. Manag. 26:100691. doi: 10.1016/j.intman.2019.100691, 41269356

[ref11] Cao S. Geok S. K. Roslan S. Qian S. Sun H. Lam S. K. . (2022). Mindfulness-based interventions for the recovery of mental fatigue: a systematic review. Int. J. Environ. Res. Public Health 19:7825. 7825. doi: 10.3390/ijerph19137825, 35805484 PMC9265434

[ref12] Carnevale J. B. Hatak I. (2020). Employee adjustment and well-being in the era of COVID-19: implications for human resource management. J. Bus. Res. 116, 183–187. doi: 10.1016/j.jbusres.2020.05.037, 32501303 PMC7241356

[ref13] Cassetta E. Monarca U. Dileo I. Di Berardino C. Pini M. (2020). The relationship between digital technologies and internationalization: evidence from Italian SMEs. Ind. Innov. 27, 311–339. doi: 10.1080/13662716.2019.1696182

[ref14] Cetindamar-Kozanoglu D. Abedin B. (2021). Understanding the role of employees in digital transformation: conceptualization of digital literacy of employees as a multi-dimensional organizational affordance. J. Enterp. Inf. Manag. 34, 1649–1672. doi: 10.1108/jeim-01-2020-0010

[ref15] Chatterjee S. Sheshadri C. Vrontis R. Giovando G. (2023). Digital workplace and organization performance: moderating role of digital leadership capability. J. Innov. Knowl. 8:100334. doi: 10.1016/j.jik.2023.100334

[ref16] Chesley N. (2014). Information and communication technology use, work intensification, and employee strain and distress. Work Employ. Soc. 28, 589–610. doi: 10.1177/0950017013500112

[ref17] Creswell J. D. (2017). Mindfulness interventions. Annu. Rev. Psychol. 68, 491–516. doi: 10.1146/annurev-psych-042716-051139 27687118

[ref18] Daniel C. Chowdhury R. M. M. I. Gentina E. (2024). Mindfulness, spiritual well-being, and sustainable consumer behavior. J. Clean. Prod. 455:142293. doi: 10.1016/j.jclepro.2024.142293

[ref19] Day A. Scott N. Kelloway E. K. (2010). “Information and communication technology: implications for job stress and employee well-being” in New developments in theoretical and conceptual approaches to job stress, 317–350. doi: 10.1108/S1479-3555(2010)0000008011

[ref20] De Vibe M. Solhaug I. Tyssen R. Friborg O. Rosenvinge J. H. Sørlie T. . (2013). Mindfulness training for stress management: a randomized controlled study of medical and psychology students. BMC Med. Educ., 13, 1–11. doi: 10.1186/1472-6920-13-107 23941053 PMC3751423

[ref21] Dén-Nagy I. (2014). A double-edged sword? A critical evaluation of the mobile phone in creating work–life balance. New Technol. Work Employ. 29, 193–211. doi: 10.1111/ntwe.12031

[ref22] Deng H. Duan S. X. Wibowo S. (2023). Digital technology-driven knowledge sharing for job performance. J. Knowl. Manag. 27, 404–425. doi: 10.1108/JKM-08-2021-0637

[ref23] Diller M. Asen M. Späth T. (2020). The effects of personality traits on digital transformation: evidence from German tax consulting. Int. J. Account. Inf. Syst. 37:100455. doi: 10.1016/j.accinf.2020.100455, 41269356

[ref24] Ertiö T. Eriksson T. Rowan W. McCarthy S. (2024). The role of digital leaders’ emotional intelligence in mitigating employee technostress. Bus. Horiz. 67, 399–409. doi: 10.1016/j.bushor.2024.03.004

[ref25] Erum H. Abid G. Contreras F. (2020). The calling of employees and work engagement: the role of flourishing at work. Bus. Manag. Econ. Eng. 18, 14–32. doi: 10.3846/bme.2020.11430

[ref26] Ferguson M. Carlson D. Boswell W. Whitten D. Butts M. M. Kacmar K. M. (2016). Tethered to work: a family systems approach linking mobile device use to turnover intentions. J. Appl. Psychol. 101:520. doi: 10.1037/apl0000075, 26653530

[ref27] Fiedler A. Casey C. Fath B. (2021). Transnational employee voice and knowledge exchange in the multinational corporation: the European company (SE) experience. Hum. Relat. 74, 1033–1059. doi: 10.1177/0018726720905351

[ref28] Fleischer J. Wanckel C. (2024). Creativity in policy capacity: organizational and individual determinants. Public Adm. Rev. 84, 218–232.2. doi: 10.1111/puar.13676

[ref9002] Gajda D. Zbierowski P. (2023). Exploring the consequences of mindfulness at work: the impact of mindful organizing on employee attitudes and behavior toward work and organization. Personnel Review, 52, 2342–2362.

[ref29] Glomb T. M. Duffy M. K. Bono J. E. Yang T. (2011). “Mindfulness at work” in Research in personnel and human resources management. eds. Joshi A. Liao H. Martocchio J. J., vol. 30 (Leeds: Emerald Group Publishing Limited), 115–157.

[ref30] Gong Y. Huang J. C. Farh J. L. (2009). Employee learning orientation, transformational leadership, and employee creativity: the mediating role of employee creative self-efficacy. Acad. Manag. J. 52, 765–778. doi: 10.5465/amj.2009.43670890

[ref31] Goyal R. Sharma H. (2024). Does well-being mediate between mindfulness and knowledge workers’ work engagement relationship? J. Knowl. Econ. 15, 4004–4023. doi: 10.1007/s13132-023-01313-w, 40479027 PMC10039441

[ref32] Gunasekara A. Zheng C. S. M. (2019). Examining the effect of different facets of mindfulness on work engagement. Employ. Relat. 41, 193–208. doi: 10.1108/ER-09-2017-0220

[ref33] Hair J. F. Jr. Anderson R. E. Tatham R. L. Black W. C. (1998). Multivariate Data Analysis. 5th Edn. Upper Saddle River, NJ: Prentice Hall.

[ref34] Hakanen J. J. Perhoniemi R. Toppinen-Tanner S. (2008). Positive gain spirals at work: from job resources to work engagement, personal initiative, and work-unit innovativeness. J. Vocat. Behav. 73, 78–91. doi: 10.1016/j.jvb.2008.01.003

[ref35] Halbesleben J. R. B. Neveu J. P. Paustian-Underdahl S. C. Westman M. (2014). Understanding the role of resources in conservation of resources theory. J. Manage. 40, 1334–1364. doi: 10.1177/0149206314527130

[ref36] Hatch N. W. Dyer J. H. (2004). Human capital and learning as a source of sustainable competitive advantage. Strateg. Manage. J. 25, 1155–1178. doi: 10.1002/smj.421

[ref37] Hendrawan S. A. Chatra A. Iman N. Hidayatullah S. Suprayitno D. (2024). Digital transformation in MSMEs: challenges and opportunities in technology management. Jurnal Informasi dan Teknologi, 141–149. doi: 10.60083/jidt.v6i2.551

[ref38] Henle C. A. Blanchard A. L. (2008). The interaction of work stressors and organizational sanctions on cyberloafing. J. Manag. Issues, 383–400. doi: 10.2307/40604617

[ref39] Henriksen D. Richardson C. Shack K. (2020). Mindfulness and creativity: implications for thinking and learning. Think. Skills Creat. 37:100689. doi: 10.1016/j.tsc.2020.100689, 32834868 PMC7395604

[ref40] Hobfoll S. E. (2001). The influence of culture, community, and the nested-self in the stress process: advancing conservation of resources theory. Appl. Psychol. 50, 337–421. doi: 10.1111/1464-0597.00062

[ref41] Hobfoll S. E. (2002). Social and psychological resources and adaptation. Rev. Gen. Psychol. 6, 307–324. doi: 10.1037/1089-2680.6.4.307

[ref42] Hobfoll S. E. Johnson R. J. Ennis N. Jackson A. P. (2003). Resource loss, resource gain, and emotional outcomes among inner city women. J. Pers. Soc. Psychol. 84, 632–643. doi: 10.1037/0022-3514.84.3.632, 12635922

[ref43] Hobfoll S. E. Wells J. D. (1998). “Conservation of resources, stress, and aging: why do some slide and some spring?” in Handbook of aging and mental health: An integrative approach, (Berlin: Springer), 121–134. doi: 10.1007/978-1-4899-0098-2_6

[ref44] Hofmann S. G. Sawyer A. T. Witt A. A. Oh D. (2010). The effect of mindfulness-based therapy on anxiety and depression: a meta-analytic review. J. Consult. Clin. Psychol. 78, 169–183. doi: 10.1037/a0018555, 20350028 PMC2848393

[ref45] Hur W. M. Shin Y. Kim J. Y. (2024). Service employees’ mindfulness and job crafting amid COVID-19: the roles of resilience, organizational health climate, and health-oriented leadership. Curr. Psychol. 43, 16979–16991. doi: 10.1007/s12144-023-04714-x, 37359638 PMC10166686

[ref46] Hutchby I. (2001). Technologies, texts and affordances. Sociology 35, 441–456. doi: 10.1177/S0038038501000219

[ref47] Huu P. T. (2023). Impact of employee digital competence on the relationship between digital autonomy and innovative work behavior: a systematic review. Artif. Intell. Rev. 56, 14193–14222. doi: 10.1007/s10462-023-10492-6, 37362897 PMC10148002

[ref48] Jan A. I. Cautisanu C. Grădinaru C. Tă nă sescu C. De Moraes G. (2022). Exploring digital literacy skills in social sciences and humanities students. Sustainability 14:2483. doi: 10.3390/su14052483

[ref49] Janse van Rensburg C. Coetzee S. A. Schmulian A. (2022). Develop digital creativity through authentic assessment. Assess. Eval. High. Educ. 47, 857–877. doi: 10.1080/02602938.2021.1968791

[ref50] Kaltenegger H. C. Becker L. Rohleder N. Nowak D. Weigl M. (2020). Association of working conditions including digital technology use and systemic inflammation among employees: study protocol for a systematic review. Syst. Rev. 9, 1–11. doi: 10.1186/s13643-020-01463-x, 32988415 PMC7523305

[ref51] Keyes C. L. M. (2020). The mental health continuum: from languishing to flourishing. J. Health Soc. Behav. 43, 207–222. 12096700

[ref52] Kil H. O'Neill D. Grusec J. E. (2021). Prosocial motivation as a mediator between dispositional mindfulness and prosocial behavior. Pers. Individ. Differ. 177:110806. doi: 10.1016/j.paid.2021.110806

[ref53] Kim M. Beehr T. A. (2020). Thriving on demand: challenging work results in employee flourishing through appraisals and resources. Int. J. Stress Manage. 27:111. doi: 10.1037/str0000135

[ref54] Kim M. Seong G. Jeon M.-J. Jung Y.-C. Lee D. (2024). The mediating effect of attentional impulsivity between mindfulness and problematic smartphone use. BMC Psychiatry 24:294. doi: 10.1186/s12888-024-05708-0, 38637786 PMC11025234

[ref55] Korzynski P. Paniagua J. Rodriguez-Montemayor E. (2019). Employee creativity in a digital era: the mediating role of social media. Manag. Decis. 58, 1100–1117. doi: 10.1108/MD-05-2018-0586

[ref56] Li C. P. Shi K. (2023). The influence of distributive justice and procedural justice on job burnout. Acta Psychol. Sin. 5, 677–684. doi: 10.3969/j.issn.1673-5218.2009.12.005

[ref57] Liang L. H. Brown D. J. Ferris D. L. Hanig S. Lian H. Keeping L. M. (2018). The dimensions and mechanisms of mindfulness in regulating aggressive behaviors. J. Appl. Psychol. 103, 281–299. doi: 10.1037/apl0000283, 29154582

[ref58] Lim V. K. G. (2002). The IT way of loafing on the job: cyberloafing, neutralizing, and organizational justice. J. Organ. Behav. 23, 675–694. doi: 10.1002/job.161

[ref59] Loucks E. B. Crane R. S. Sanghvi M. A. Montero-Marin J. Proulx J. Brewer J. A. . (2022). Mindfulness-based programs: why, when, and how to adapt? Global Adv. Health Med. 11:21649561211068805. doi: 10.1177/21649561211068805, 35127272 PMC8811951

[ref60] Lyddy C. J. Good D. J. Bolino M. C. Thompson P. S. Stephens J. P. (2021). The costs of mindfulness at work: the moderating role of mindfulness in surface acting, self-control depletion, and performance outcomes. J. Appl. Psychol. 106, 1921–1938. doi: 10.1037/apl0000863, 33570968

[ref61] MacDonald W. (2003). The impact of job demands and workload on stress and fatigue. Aust. Psychol. 38, 102–117. doi: 10.1080/00050060310001707107

[ref62] Marion T. J. Fixson S. K. (2021). The transformation of the innovation process: how digital tools are changing work, collaboration, and organizations in new product development. J. Prod. Innov. Manag. 38, 192–215. doi: 10.1111/jpim.12547

[ref63] Marsh E. Vallejos E. P. Spence A. (2022). The digital workplace and its dark side: an integrative review. Comput. Human Behav. 128:107118. doi: 10.1016/j.chb.2021.107118

[ref64] Montreuil V. L. Dextras-Gauthier J. Gilbert M. H. Dima J. Boulet M. (2022). Remote work during the COVID-19 pandemic: how do digital technology use affect mental fatigue, psychological distress, and well-being? Saf. Health Work 13:S166. doi: 10.1016/j.shaw.2021.12.1271

[ref65] Mulki J. P. Jaramillo F. Locander W. B. (2006). Emotional exhaustion and organizational deviance: can the right job and a leader's style make a difference? J. Bus. Res. 59, 1222–1230. doi: 10.1016/j.jbusres.2006.09.001

[ref66] Ngo L. V. Nguyen N. P. Lee J. Andonopoulos V. (2020). Mindfulness and job performance: does creativity matter? Australas. Mark. J. 28, 117–123. doi: 10.1016/j.ausmj.2019.12.003

[ref67] Nikou S. De Reuver M. Mahboob Kanafi M. (2022). Workplace literacy skills: how information and digital literacy affect the adoption of digital technology. J. Doc. 78, 371–391.

[ref68] Oldham G. R. Cummings A. (1996). Employee creativity: personal and contextual factors at work. Acad. Manag. J. 39, 607–634. doi: 10.2307/256657

[ref69] Oldham G. R. Da Silva N. (2015). The impact of digital technology on the generation and implementation of creative ideas in the workplace. Comput. Hum. Behav. 42, 5–11. doi: 10.1016/j.chb.2013.10.041

[ref70] Orlandi L. B. Pocek J. Kraus S. Zardini A. Rossignoli C. (2024). Digital workers’ stress: the role of digital creativity in future jobs. J. Innov. Knowl. 9:100492.00492.

[ref71] Pfaffinger K. F. Reif J. A. Spieß E. Berger R. (2020). Anxiety in a digitalized work environment. Gr Interakt Org 51, 25–35. doi: 10.1007/s11612-020-00502-4

[ref72] Pink S. Lingard H. Harley J. (2017). Refiguring creativity in virtual work: the digital-material construction site. New Technol. Work Employ. 32, 12–27. doi: 10.1111/ntwe.12075

[ref73] Podsakoff P. M. MacKenzie S. B. Lee J. Y. Podsakoff N. P. (2003). Common method biases in behavioral research: a critical review of the literature and recommended remedies. J. Appl. Psychol. 88, 879–903. doi: 10.1037/0021-9010.88.5.879, 14516251

[ref74] Polly D. Martin F. Guilbaud T. C. (2021). Examining barriers and desired supports to increase faculty members’ use of digital technologies: perspectives of faculty, staff, and administrators. J. Comput. High. Educ. 33, 135–156. doi: 10.1007/s12528-020-09259-7

[ref75] Porath C. Spreitzer G. Gibson C. Garnett F. G. (2012). Thriving at work: toward its measurement, construct validation, and theoretical refinement. J. Organ. Behav. 33, 250–275. doi: 10.1002/job.756

[ref76] Preacher K. J. Hayes A. F. (2008). Asymptotic and resampling strategies for assessing and comparing indirect effects in multiple mediator models. Behav. Res. Methods 40, 879–891. doi: 10.3758/BRM.40.3.879, 18697684

[ref77] Puranik H. Koopman J. Vough H. C. (2019). Pardon the interruption: an integrative review and future research agenda for research on work interruptions. J. Manage., 2019,46, 806–842. –842.

[ref78] Saniuk S. Caganova D. Saniuk A. (2023). Knowledge and skills of industrial employees and managerial staff for the implementation of industry 4.0. Mobile Netw. Appl. 28, 220–230.

[ref79] Siyal A. W. Hongzhuan C. Khan I. Chen G. (2023). Employees' resistance and turnover intentions to the use of digital technologies: from the perspective of leadership. Front. Psychol. 14:1222889. doi: 10.3389/fpsyg.2023.1222889 37599731 PMC10436076

[ref80] Sonnentag S. Kuttler I. Fritz C. (2010). Job stressors, emotional exhaustion, and need for recovery: a multi-source study on the benefits of psychological detachment. J. Vocat. Behav. 76, 355–365. doi: 10.1016/j.jvb.2009.06.005

[ref81] Stich J. F. Farley S. Cooper C. Tarafdar M. (2015). Information and communication technology demands: outcomes and interventions. J. Organ. Eff. People Perform. 2, 327–345. doi: 10.1108/JOEPP-09-2015-0031

[ref82] Tang N. Han L. Yang P. Zhao Y. Zhang H. (2019). Are mindfulness and self-efficacy related to presenteeism among primary medical staff? A cross-sectional study. Int. J. Nurs. Sci. 6, 182–186. doi: 10.1016/j.ijnss.2019.03.004, 31406889 PMC6608653

[ref83] Tekic Z. Koroteev D. (2019). From disruptively digital to proudly analog: a holistic typology of digital transformation strategies. Bus. Horiz. 62, 683–693. doi: 10.1016/j.bushor.2019.07.002

[ref84] Tortorella G. L. Fogliatto F. S. Cauchick-Miguel P. A. Kurnia S. Jurburg D. (2021). Integration of industry 4.0 technologies into total productive maintenance practices. Int. J. Prod. Econ. 240:108224. doi: 10.1016/j.ijpe.2021.108224

[ref85] Verhaeghen P. (2021). Mindfulness as attention training: Meta-analyses on the links between attention performance and mindfulness interventions, long-term meditation practice, and trait mindfulness. Mindfulness 12, 564–581. doi: 10.1007/s12671-020-01532-1

[ref86] Vermesan O. Friess P. (2022). Digitising the industry, the internet of things connects the physical, digital, and virtual worlds. Boca Raton: CRC Press.

[ref9001] Vodă A. I. Cautisanu C. Grădinaru C. Tănăsescu C. de Moraes G. H. S. M. (2022). Exploring digital literacy skills in social sciences and humanities students. Sustainability, 14:2483.

[ref87] Walsh M. M. Carleton E. L. Hancock A. J. Arnold K. A. (2022). Mindfulness and stereotype threat in social media: unexpected effects for women 's leadership aspirations. Gend. Manag. 37, 535–548. doi: 10.1108/GM-11-2020-0341

[ref88] Wang X. Gu Y. Ahmad M. Xue C. (2022). The impact of digital capability on manufacturing company performance. Sustainability 14:6214. doi: 10.3390/su14106214

[ref89] Wang B. Liu Y. Parker S. K. (2020). How does the use of information communication technology affect individuals? A work design perspective. Acad. Manage. Ann. 14, 695–725. doi: 10.5465/annals.2018.0127

[ref9003] Welp A. Meier L. L. Manser T. (2015). Emotional exhaustion and workload predict clinician-rated and objective patient safety. Front. Psychol. 5:1573. 25657627 10.3389/fpsyg.2014.01573 PMC4302790

[ref90] Yan H. Huang X. Qian C. Chen B. (2024). When lonely employees are productive: an intervention study on workplace mindfulness and job autonomy. Curr. Psychol. 43, 23036–23052. doi: 10.1007/s12144-024-06036-y, 41268434

[ref91] Yang C. Huang Q. Li Z. Liu K. Hu F. (2017). Big data and cloud computing: innovation opportunities and challenges. Int. J. Digit. Earth 10, 13–53. doi: 10.1080/17538947.2016.1239771

[ref92] Zahoor N. Christofi M. Nwoba A. C. Donbesuur F. Miri D. (2024). Operational effectiveness in post-pandemic times: examining the roles of digital technologies, talent management, and employee engagement in manufacturing SMEs. Prod. Plan. Control 35, 1625–1638. doi: 10.1080/09537287.2022.2147863

[ref93] Zheng X. Ni D. Liu X. Liang L. H. (2023). Workplace mindfulness: multidimensional model, scale development and validation. J. Bus. Psychol. 38, 777–801. doi: 10.1007/s10869-022-09814-2

[ref94] Zivnuska S. Kacmar K. M. Ferguson M. Carlson D. S. (2016). Mindfulness at work: resource accumulation, well-being, and attitudes. Career Dev. Int. 21, 106–124. doi: 10.1108/CDI-06-2015-0086

